# The Microtubule-Modulating Drug Epothilone D Alters Dendritic Spine Morphology in a Mouse Model of Mild Traumatic Brain Injury

**DOI:** 10.3389/fncel.2018.00223

**Published:** 2018-07-30

**Authors:** Jyoti A. Chuckowree, Zhendan Zhu, Mariana Brizuela, Ka M. Lee, Catherine A. Blizzard, Tracey C. Dickson

**Affiliations:** ^1^Menzies Institute for Medical Research, University of Tasmania, Hobart, TAS, Australia; ^2^Centre for Neuroscience, School of Medicine, Flinders University, Adelaide, SA, Australia; ^3^The Florey Institute of Neuroscience and Mental Health, Parkville, VIC, Australia

**Keywords:** traumatic brain injury, fluid percussion injury, neuroplasticity, microtubule stabilization, epothilone D, dendritic spine, cortical projection neuron, mushroom spine

## Abstract

Microtubule dynamics underpin a plethora of roles involved in the intricate development, structure, function, and maintenance of the central nervous system. Within the injured brain, microtubules are vulnerable to misalignment and dissolution in neurons and have been implicated in injury-induced glial responses and adaptive neuroplasticity in the aftermath of injury. Unfortunately, there is a current lack of therapeutic options for treating traumatic brain injury (TBI). Thus, using a clinically relevant model of mild TBI, lateral fluid percussion injury (FPI) in adult male Thy1-YFPH mice, we investigated the potential therapeutic effects of the brain-penetrant microtubule-stabilizing agent, epothilone D. At 7 days following a single mild lateral FPI the ipsilateral hemisphere was characterized by mild astroglial activation and a stereotypical and widespread pattern of axonal damage in the internal and external capsule white matter tracts. These alterations occurred in the absence of other overt signs of trauma: there were no alterations in cortical thickness or in the number of cortical projection neurons, axons or dendrites expressing YFP. Interestingly, a single low dose of epothilone D administered immediately following FPI (and sham-operation) caused significant alterations in the dendritic spines of layer 5 cortical projection neurons, while the astroglial response and axonal pathology were unaffected. Specifically, spine length was significantly decreased, whereas the density of mushroom spines was significantly increased following epothilone D treatment. Together, these findings have implications for the use of microtubule stabilizing agents in manipulating injury-induced synaptic plasticity and indicate that further study into the viability of microtubule stabilization as a therapeutic strategy in combating TBI is warranted.

## Introduction

In popular media, mild traumatic brain injury (mTBI) has been referred to as a ‘silent epidemic.’ Indeed, in a majority of mTBI cases there is distinct absence of clear structural damage alongside normal neuroimaging (Iverson, 2005, 2010; Belanger et al., 2007; Jagoda et al., 2009; Gao and Chen, 2011; Smith et al., 2013). Nevertheless, subtle perturbations in brain structure likely evoke an insidious cascade of evolving widespread damage to neural circuitry, thought to culminate in long-term and ongoing neurological impairment and associated problems (Büki and Povlishock, 2006; Farkas and Povlishock, 2007; Blyth and Bazarian, 2010; Iverson, 2010; Wang and Ma, 2010; Meaney and Smith, 2011; Johnson et al., 2012a; Hill et al., 2016). This typically incorporates widespread axonal perturbation, known as traumatic axonal injury, throughout the parenchyma and particularly in the white matter tracts. This injury may include changes in the somato-dendritic compartment such as somal atrophy, distorted dendritic arbor geometry and loss of complexity, and decreased dendritic spine density (Chen et al., 2003, 2010; Stone et al., 2004; Spain et al., 2010; Gao and Chen, 2011; Gao et al., 2011; Greer et al., 2011; Campbell et al., 2012).

Data from experimental models shows that mTBI characteristically generates sparse microscopic damage, whereby neural circuits are rendered dysfunctional but not destroyed (Iverson, 2005; DeKosky and Ikonomovic, 2010; Shultz et al., 2016). This highlights an important target for therapeutic intervention. Microtubule disruption and loss is a key ultrastructural hallmark of neuronal injury (Maxwell and Graham, 1997; Tang-Schomer et al., 2012). Moreover, microtubule dynamics are fundamental to a multitude of neuro-glial responses in the aftermath of injury (Chuckowree and Vickers, 2003; Tang-Schomer et al., 2012; Brizuela et al., 2015). Thus, manipulating microtubules provides a novel multi-target approach for intervening in these processes (Brunden et al., 2012; Baas and Ahmad, 2013; Dent, 2016). Of note, microtubule-stabilizing agents of the taxane and epothilone families are used chemotherapeutically at high doses to block the growth of cancerous cells (Goodin, 2004; Michaud, 2009; Zhao et al., 2009; Khrapunovich-Baine et al., 2011). Accumulating data derived from a variety of experimental neural injury and disease paradigms reveals that when used at low doses these drugs have a range of beneficial effects, including dampening detrimental gliotic responses, preventing synapse loss and enhancing adaptive neuronal alterations as well as preserving cognitive and motor functions (Moscarello et al., 2002; Zhang et al., 2005; Andrieux et al., 2006; Ertürk et al., 2007; Brunden et al., 2010, 2011, 2012; Hellal et al., 2011; Sengottuvel et al., 2011; Barten et al., 2012; Tang-Schomer et al., 2012; Baas and Ahmad, 2013; Cartelli et al., 2013; Hur and Lee, 2014; Popovich et al., 2014; Brizuela et al., 2015; Cross et al., 2015; Ruschel et al., 2015; Jang et al., 2016; Penazzi et al., 2016).

The epothilones promote microtubule formation and stabilization, and inhibit microtubule depolymerization (Altmann et al., 2000; Chen et al., 2008). With particular relevance to the brain, epothilones are more water soluble than their taxane counterparts, are blood–brain barrier penetrant, and are retained in the central nervous system for several days after administration (Andrieux et al., 2006; Browne et al., 2011). Importantly, the efficacy of epothilones as a therapeutic strategy in the context of brain injury remains to be elucidated. To address this shortfall we explored the effect of peripherally administered epothilone D following a single mTBI using the clinically relevant lateral fluid percussion brain injury (FPI) model (Thompson et al., 2005). To visualize discrete alterations in the somato-dendritic and axonal compartments of layer 5 cortical excitatory projections neurons we used the Thy1-YFPH mouse, which revealed exquisite neuronal sub-structure (Feng et al., 2000).

## Materials and Methods

### Breeding and Genotyping of Thy1-YFPH Transgenic Mice

All experimental procedures involving animals were approved by the Animal Ethics Committee of the University of Tasmania (ethics approval number A0011076) and are in accordance with the Australian Code of Practice for the Care and Use of Animals for Scientific Purposes. Animals were housed in standard conditions (20°C, 12 h/12 h light/dark cycle) with access to food and water *ad libitum* and monitored daily for signs of stress and illness. Thy1-YFPH line mice [B6.Cg-Tg(Thy1-YFP)HJrs/J, stock number 003782] were obtained from the Jackson Laboratory (Bar Harbor, ME, United States) and maintained as a heterozygous colony. In these animals YFP is expressed under the control of the neuron-specific Thy1 promoter in ∼80% of layer 5 and 2/3 neocortical pyramidal neurons (Feng et al., 2000). Ear punches were taken at weaning (4 weeks) to determine inheritance of the YFP transgene. Tissue was mounted on a glass slide and examined with the 488 nm filter on a Leica DM LB2 microscope (Leica Microsystems Pty Ltd., North Ryde, NSW, Australia). Animals carrying the YFP transgene were identified as possessing YFP-positive (YFP+) axons within their ear clips.

### Surgical Preparation

Animals were prepared in groups of four per day. Two animals received FPI while two were sham-operated. Mice were subjected to lateral FPI using an established protocol (Carbonell et al., 1998; Lifshitz et al., 2007; Alder et al., 2011). Briefly, adult male YFP-H mice (10–12 weeks, 25–30 g; *n* = 12 FPI/brain-injured, *n* = 12 sham-operated) were anesthetised in a pre-charged induction chamber containing 5% isoflurane (Isoflo, Abbot Australasia Pty Ltd., Botany, NSW, Australia) in 100% O_2._ Mice were removed from the induction chamber, pre-emptive analgesia, temgesic (buprenorphine hydrochloride, 0.1 mg/kg; Reckitt Benckiser, West Ryde, NSW, Australia), was administered subcutaneously and the fur covering the scalp was removed. Mice were placed on a homeothermic blanket (Stoelting, Wood Dale, IL, United States) to maintain body temperature at 37°C during surgery and stabilized in a stereotaxic frame (Narishige, Tokyo, Japan) equipped with a nose cone to maintain anesthesia (1–2% isoflurane in 100% O_2_). The scalp was cleaned with betadine (Sanofi-aventis Consumer Healthcare, Virginia, QLD, Australia) and 70% ethanol and the topical anesthetic bupivicaine (Bupivicaine hydrochloride, 50 μl 0.25% in sterile saline; Pfizer, West Ryde, NSW, Australia) was administered under the scalp. A midline incision was made to expose the skull from bregma to lambda. The skin was retracted and the fascia covering the skull was removed. A 3.0 mm circular craniectomy was made 2.0 mm posterior and 2.5 mm lateral to bregma on the right hand side of the skull over the somatosensory cortex via manual trephination with a pin vice equipped with a 2.7 mm trephine drill bit (AgnTho’s, Lidingo, Sweden). The underlying dura was left intact. An injury-hub was constructed over the craniectomy – a sterile Luer-Loc syringe hub was cut from a 22-gauge needle, fixed over the craniectomy using Loctite cyanoacrylate (Henkel Australia, Sydney, NSW, Australia), secured to the skull using Paladur dental acrylic (Heraeus Dental Science, Villebon, France), filled with sterile saline and capped with a male Luer-Loc fitting. Pre-, peri-, and post-surgical monitoring was performed to determine respiratory rate, confirm absence of reflexes and monitor mucous membranes/capillary refill time. Following injury-hub placement animals were removed from the stereotaxic frame and allowed to recover in a warmed cage until fully ambulatory (30–60 min) and then placed back in their home cage.

### Lateral Fluid Percussion Brain Injury and Drug Treatment

Two hours following application of the injury-hub, once mice had been fully ambulatory for over an hour, each animal was re-anesthetised in a pre-charged induction chamber containing 5% isoflurane in 100% O_2_. Following induction of anesthesia, the animal was removed from the induction chamber, the cap was removed from the injury hub and the hub was re-filled with sterile saline and attached to the FP302 Fluid Percussion Device (AmScien Instruments, Richmond, VA, United States) via a 30 cm spacing tube filled with sterile water. The animal was placed on a heated pad and once a normal pattern of breathing resumed, but prior sensitivity to stimulation, an injury of mild severity (1.5 ± 0.1 atmospheres) was delivered to the intact dura by releasing device’s pendulum onto a fluid filled piston, causing transient displacement and deformation of the dura and underlying brain. A transducer incorporated into the device measured the pulse pressure and the peak pressure was recorded within the software. Following injury, animals were placed on their back and visually monitored for recovery of spontaneous breathing. Additionally, the time taken for animals to recover the righting reflex was recorded as a measure of transient unconsciousness/loss of consciousness. Following injury, we did not record any convulsions, mortalities or other complications. Sham-operated animals underwent identical procedures to FPI animals, however, the pendulum was not released. Mice were re-anesthetised, the injury hub was removed and the incision sutured. Immediately following suturing, animals were administered with either epothilone D (2 mg/kg, i.p.; Anita Laboratories, Hangzhou, China; *n* = 6 FPI and *n* = 6 sham-operated) or vehicle (equivalent volume DMSO; *n* = 6 FPI and *n* = 6 sham-operated). Mice received drug/vehicle treatment within 20 min following completion application of the FPI/Sham-operation. Animals were placed in a heated cage and monitored during the recovery period until fully ambulatory (30–60 min), prior to return to their home cage.

### Immunohistochemistry

At 1 week post-injury/sham-operation, mice were intraperitoneally injected with a terminal dose of sodium pentobarbital (300 mg/kg; Troy Laboratories Pty Ltd., Smithfield, NSW, Australia) and transcardially perfused with 4% paraformaldehyde in 0.1 M phosphate buffer. Brains were post-fixed *in vivo* for 24 h at 4°C. Each brain was removed from the skull, embedded in 5% agarose in 0.01 M phosphate buffered saline (PBS) and free-floating coronal sections (50 μm) were cut using a Leica VT1000S vibratome (Leica Biosystems Australia Pty Ltd., Mount Waverly, VIC, Australia) to incorporate the entire injury impact site as well as 0.5–1.0 mm anterior and posterior to this. Sections were serially collected into Costar 24-well culture plates (Corning Life Sciences, New York, NY, United States) containing 0.01M PBS and 0.02% sodium azide and stored until required.

To perform immunohistochemistry, every sixth section from each brain was moved into a fresh culture plate well to represent the injury site. Prior to immunohistochemistry, sections were rinsed in three washes of 0.01 M PBS. Sections then underwent immunofluorescence labeling for glial fibrillary acidic protein (GFAP). Briefly, sections were incubated in rabbit anti-GFAP (1:2000; DAKO, Z0334, Glostrup, Denmark) in diluent (0.01 M PBS with 0.03% Triton X-100) at 4°C for ∼20 h, washed, incubated in goat anti-rabbit Alexa 568 (1:1000; Invitrogen BRL, Life Technologies, Grand Island, NY, United States) and DAPI (1:6000; Invitrogen, D3571) in 0.01M PBS for 1.5 h, washed and mounted serially onto slides (Livingstone International Pty Ltd., Rosebery, NSW, Australia) with Permafluor mounting media (Thermo Scientific, Scoresbury, VIC, Australia).

### Microscopy and Image Analysis

Images were collected with an UltraVIEW spinning disk confocal microscope running Volocity Software (PerkinElmer Pty Ltd., Glen Waverley, VIC, Australia), equipped with a 20×/0.5 air, 40×/0.95 air and Plan Apo 60×/1.20 water objective (Nikon, New York, NY, United States). For quantitation of cortical thickness, YFPH cell number and size and degenerated/dystrophic axonal bulb number and size (in the internal and external capsules) the microscope was configured to capture large stitched images of the upper hemispheric quadrant of each brain (20 μm z-stacks, 1 μm slices) with the 20× objective from three representative sections throughout the injury, designated middle, anterior, and posterior representing the middle section (first appearance of two blades of the dentate gyrus) as well as the section 300 μm anterior and 300 μm posterior to this. Using ImageJ freeware (Schneider et al., 2012) the cortex, external and internal capsule were traced in each of the three sections and within these anatomical boarders the individual YFPH+ cells in the cortex and axonal bulbs/dystrophic neurites in the external and internal capsules were traced for quantitation of size and density. To determine the axonal degeneration index (degenerating/beaded YFPH+ axons) single 40× magnification image stacks (20 μm z-stacks, 1 μm slices) were collected from the same three sections as used for the prior analysis. Images for the external capsule were captured from the white matter on the medio-lateral boarder of the lateral ventricle and those for the internal capsule were captured half way between the dorsal and ventral boarder of the internal capsule. For quantification of astrocyte activation (percent area occupied by GFAP expressing astrocytes), 20× single image stacks (20 μm z-stacks, 1 μm slices) were collected from the same regions used for analysis of axonal denegation, in addition to layer 2/3 of the cortex directly under the impact site. Analysis of percentage area GFAP and YFPH+ axonal degeneration was performed using ImageJ freeware. For dendritic spine analysis, image stacks were captured with the 60× water objective (0.2 μm slices). Image stacks were collected from layer 4/5 directly under the injury site. These included the image in the middle of the impact site as well as an image medial and lateral to this. All layer 5 apical dendrite obliquely projecting branches were traced from each stack and the spines contained on these dendrites were traced in Neurolucida (MBF Biosciences, Williston, VT, United States). Morphology data was generated by allocating each spine to one of three categories, mushroom (prominent head, thin neck), stubby (greater width than length), and thin (greater length than width). Changes in dendritic spine density, length, and morphology were determined by loading Neurolucida data files into Neurolucida Explorer^TM^ (MBF Biosciences).

### Statistical Analysis

Data was analyzed (and graphs created) in GraphPad Prism (version 6.0, La Jolla, CA, United States) using unpaired *t*-tests with Welch’s correction, or one- or two-way analysis of variance (ANOVA) followed by Tukey’s multiple comparison test. Averaged values were expressed as means ± standard error of the mean (SEM). A *p*-value of <0.05, designated ^∗^, was considered statistically significant. Figures were prepared in Adobe Illustrator CS6 (version 16.0.0, Adobe Systems, San Jose, CA, United States).

## Results

### A Single Mild Lateral FPI Caused a Transient Loss of Consciousness in the Absence of Overt Morphological Change

Adult male Thy1-YFPH mice received a single mild lateral FPI or sham-operation followed by epothilone D or vehicle treatment and were perfused 7 days later. In brain-injured mice the acute post-injury period was characterized by a transient loss of consciousness, including a short interval of apnoea (0.21 ± 0.04 min) and a significant delay in the righting reflex (3.64 ± 1.63 min in brain-injured relative to 0.19 ± 0.13 min in sham-operated animals; *p* < 0.0001, unpaired *t*-test with Welch’s correction). By 7 days post-injury, there were no gross morphological alterations in the brain following a single mild lateral FPI: cortical thickness, as well as the number and somal size of cortical layer 5 YFP+ projection neurons, remained unchanged (**Table 1**, *p* > 0.05 for all comparisons). Moreover, there was no change in axonal number (axons per field of view) in the external and internal capsules, or the length of apical oblique dendrites (total length of dendrite per field of view) of layer 5 pyramidal neurons (**Table 1**). Epothilone D treatment did not significantly (*p* > 0.05 for all comparisons) affect any of these parameters, with cortical thickness, number and size of YFP+ cells, number of YFP+ axons in both the external and internal capsules and length of YFP+ apical oblique dendrites remaining unaltered following peripherally administered epothilone D (**Table 1**).

**Table 1:** Histological analyses comparing brain-injured and sham-operated animals following drug treatment.

	Sham veh	FPI veh	Sham Epo	FPI Epo
Cortical thickness (mm)	1.11 ± 0.06	1.07 ± 0.05	1.10 ± 0.05	1.08 ± 0.05
YFP+ cell density (per mm^2^)	36.67 ± 8.85	38.50 ± 10.43	35.67 ± 8.31	36.4 ± 4.72
YFP+ cell size (μm^2^)	159 ± 14.11	158.5 ± 13.4	159.67 ± 12.03	161.6 ± 6.54
Number axons Ext cap (per fov)	105.17 ± 24.12	101.5 ± 25.49	110.33 ± 26.46	83.83 ± 35.61
Number axons Int cap (per fov)	166.5 ± 20.81	164.33 ± 27.86	167.33 ± 35.24	161 ± 40.33
Dendrite length (μm)	1.27 ± 0.57	1.12 ± 0.51	1.08 ± 0.38	1.10 ± 0.37

*Mice were exposed to a lateral fluid percussion injury or sham-operation and treated with epothilone D (2 mg/kg) or vehicle. Histological analyses confirmed there were no significant differences (p > 0.05) between groups for any of the parameters assessed. Ext cap, external capsule; int cap, internal capsule; fov, field of view; veh, vehicle; Epo, epothilone D; FPI, lateral fluid percussion injury.*

### Epothilone D Treatment Altered Dendritic Spine Length, density, and Morphology

**FIGURE 1:**
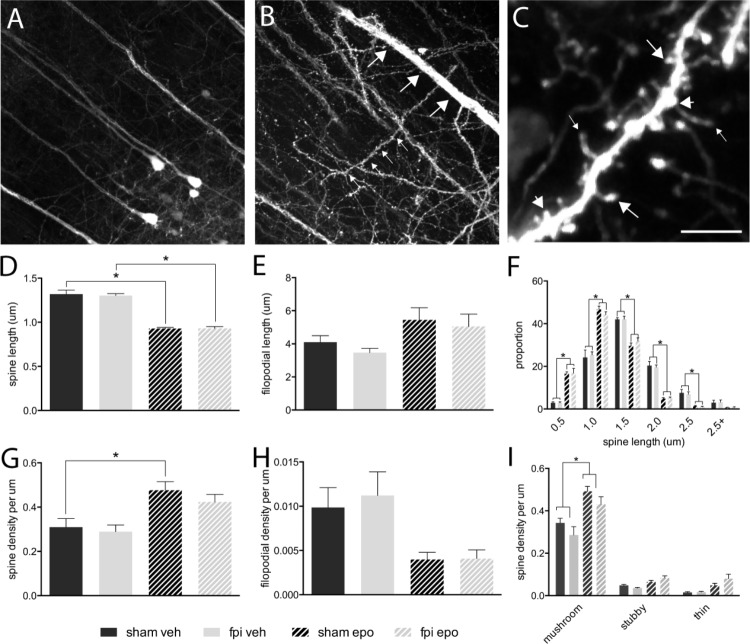
Dendritic spine alterations in layer 5 pyramidal neurons in the adult mouse brain following mild lateral fluid percussion brain injury and epothilone D treatment. Analysis was performed on layer 5 YFP-expressing neuron **(A)** apical oblique dendrites, which are laterally projecting spiney dendrites in layer 4/5 (small arrows, **B**) protruding from layer 5 neuron apical dendrites (large arrows, **B**). For analysis, dendritic protrusions/spines were classified both by length (spines <2.5 μm, **D**,**F**; filopodia > 2.4 μm, **E**,**G**) and morphology (**C**,**I**): either mushroom (large arrows in **C**), stubby (short arrows in **C**), or thin (small arrows in **C**). Epothilone D treatment resulted in a significant decrease in spine length in both brain-injured and sham-operated animals, relative to their vehicle-treated controls **(D)**, whereas filopodial length was unaffected **(E)**. Binning the data by length revealed that epothilone D treatment resulted in a significantly higher proportion of shorter spines (up to 1.5 μm) and significantly lower proportion of longer spines (1.5–2.5 μm) **(F)**. Moreover, epothilone D treatment significantly increased the density of dendritic spines in sham-operated, but not brain-injured animals **(G)**, while filopodial density was unaffected **(H)**. Analysis by morphological sub-class showed that the epothilone D treatment resulted in increased density of mushroom spines in both brain-injured and sham-operated animals relative to their vehicle-treated counterparts, while density of stubby and thin spines was unaffected **(I)**. Data are presented as mean ± SEM and were analyzed by one-way **(D,E,G,H)** or two-way **(F,I)** ANOVA, followed by Tukey’s multiple comparison test. A *p*-value of < 0.05 was considered significant (^∗^). Scale bar **(A)** = 85 μm; **(B)** = 45 μm; **(C)** = 3.5 μm. Yellow fluorescent protein (YFPH); sham-operated, vehicle-treated (sham veh); fluid percussion injury, vehicle-treated (fpi veh), sham-operated, epothilone D-treated (sham epo); fluid percussion injury, epothilone D-treated (fpi epo).

Dendritic spines were analyzed from radially projecting/oblique branches of layer 5 projection neuron apical dendrites at the layer 4/5 boarder (**Figures 1A,B**). All major morphological spine classes (mushroom, stubby, thin) were represented in all mouse groups (**Figure 1C**). Initial analysis segregated the total spine population by length – spines (<2.5 μm) and filopodia (>2.5 μm) (Hering and Sheng, 2001) – and revealed a significant decrease (*p* < 0.05) in average spine length (**Figure 1D**), but not filopodial length (**Figure 1E**), in response to epothilone D treatment relative to vehicle treatment in both brain-injured and sham-operated animals. Furthermore, binning the spines by length showed that epothilone D treatment resulted in a significantly higher proportion (*p* < 0.05) of shorter (<1.5 μm) spines and significantly lower proportion (*p* < 0.05) of longer spines (1.5–2.5 μm) (**Figure 1F**). With respect to density, spine density was significantly increased (*p* < 0.05) in sham-operated, but not brain-injured animals (**Figure 1G**) in response to epothilone D treatment, whereas filopodial density was unaltered in both sham-operated and brain-injured animals (**Figure 1H**). Analysis of morphological sub-class revealed a significant increase (*p* < 0.05) specifically in mushroom spines in both brain-injured and sham-operated animals in response to epothilone D treatment (**Figure 1I**).

### Axonal Degeneration in the External and Internal White Matter Tracts Was a Major Feature of the Injured Brain and Was Not Altered by Epothilone D Treatment

**FIGURE 2:**
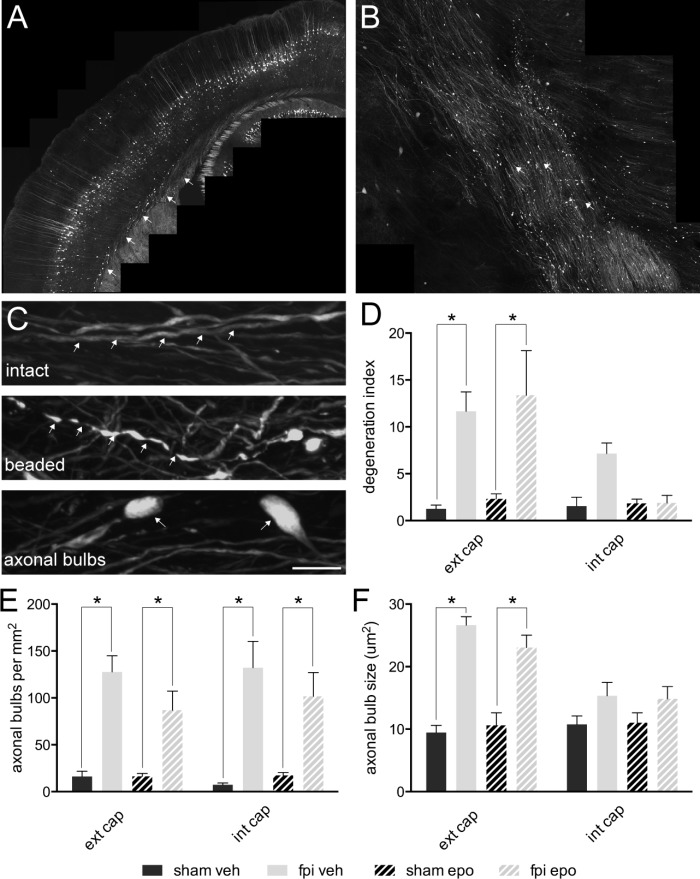
Axonal alterations in major white matter tracts in the adult mouse brain following mild lateral fluid percussion brain injury and epothilone D treatment. Mild lateral fluid percussion injury to the adult mouse brain generated a stereotypical and widespread pattern of axonal damage in the external **(A)** and internal **(B)** capsule white matter tracts. At 7 days post-injury the majority of axons remained intact (arrows, upper panel in **C**), while a proportion showed a degenerating, beaded morphology (arrows, middle panel in **C**) or a degenerated, fragmented and disconnected ‘axonal bulb’ morphology (arrows, lower panel in **C**). The proportion of beaded axons (out of the total axon population) was significantly increased in the ipsilateral external capsule of brain-injured animals relative to their sham-operated counterparts, but was unaltered in the ipsilateral internal capsule **(D)**. Analysis of axonal bulbs indicated that there were significantly more axonal bulbs in both the external and internal capsules of brain-injured animals relative to their sham-operated counterparts **(E)**. Moreover, axonal bulbs were significantly larger in the external, but not internal, capsule of brain injured animals relative to their sham-operated counterparts **(F)**. Data are presented as mean ± SEM and were analysed by two-way ANOVA **(D–F)** followed by Tukey’s multiple comparison test. A *p*-value < 0.05 was considered significant (^∗^). Scale bar **(A)** = 400 μm; **(B)** = 125 μm; **(C)** = 10 μm. Sham-operated, vehicle-treated (sham veh); fluid percussion injury, vehicle-treated (fpi veh); sham-operated, epothilone D-treated (sham epo); fluid percussion injury, epothilone D-treated (fpi epo); ipsilateral external capsule (ext cap); ipsilateral internal capsule (int cap).

By 7 days post-injury, a single mild lateral FPI had generated a distinct pattern of ipsilateral axonal damage throughout the external (**Figure 2A**) and internal (**Figure 2B**) capsule white matter tracts. Although the majority (85–90%) of YFP expressing axons remained intact, a significant proportion (*p* < 0.05) of axons showed a degenerating, beaded morphology or a disconnected, degenerated and bulbar axonal fragment morphology within the ipsilateral external capsule of brain-injured relative to sham-operated animals (**Figures 2C,D**). These axonal changes after mild FPI did not reach significance in the ipsilateral internal capsule (**Figure 2D**) or contralateral external capsule (not shown). Further analysis of the axonal response to mild FPI revealed that number and size of degenerated/dystrophic bulbar axonal fragments was significantly increased (*p* < 0.05) in the ipsilateral external capsule (**Figures 2E,F**). Moreover, degenerated axonal bulb number, but not size, was significantly increased (*p* < 0.05) in the ipsilateral internal capsule (**Figures 2E,F**) after mild FPI. Interestingly, epothilone D treatment did not alter any aspect of the axonal response to mild FPI (**Figures 2D–F**).

### Mild Lateral FPI Evoked a Limited Ipsilateral Astrogliotic Response That Was Not Influenced BY Epothilone D Treatment

Astrocyte activation was quantitated as the area occupied by GFAP immunoreactive profiles at 7 days post-injury. GFAP was significantly increased (*p* < 0.05) in the ipsilateral vs. contralateral cortex of all mice (sham-operated vehicle treated ipsilateral cortex, 6.20 ± 0.68% vs. sham-operated vehicle treated contralateral cortex, 2.98 ± 0.37%; brain-injured vehicle treated ipsilateral cortex, 6.13 ± 0.79% vs. brain-injured vehicle treated contralateral cortex, 3.23 ± 0.54%; sham-operated epothilone treated ipsilateral cortex, 5.91 ± 0.64% vs. sham-operated epothilone treated contralateral cortex, 5.61 ± 0.32%; brain-injured epothilone treated ipsilateral cortex, 5.61 ± 0.32% vs. brain-injured epothilone treated contralateral cortex, 2.87 ± 0.25%), indicating that the craniectomy itself generated mild astroglial activation. More interestingly, GFAP immunoreactive profiles (as measured by percentage area occupied by GFAP immunoreactivity) were significantly increased in the external capsule of brain-injured animals (vehicle-treated 5.79 ± 0.49%; epothilone-treated 5.20 ± 0.55%) relative to their sham-operated (vehicle-treated 3.58 ± 0.05%; epothilone-treated 3.63 ± 0.53%) controls; however, this change did not extend to the internal capsule.

## Discussion

Despite the absence of overt structural damage and the presence of normal neuroimaging in a majority of mTBI cases, subtle widespread and progressing damage within neural circuits is thought to underlie the development, and potentially ongoing evolution, of impairments in neurosensory, cognitive, psychosocial, and physical function (Iverson, 2005, 2010; Farkas and Povlishock, 2007; Dean and Sterr, 2013; McMahon et al., 2014; Hill et al., 2016; Shultz et al., 2016; Wang and Li, 2016; Wilde et al., 2016; de Koning et al., 2017). The cellular alterations contributing to pathology in the aftermath of mTBI are not fully understood and there exists no treatment to halt or reverse the damage to neural circuits. Thus, we investigated the discrete subcellular reactions of axonal and somato-dendritic compartments following transient structural brain injury and epothilone D-induced microtubule stabilization. We should note that this study represents a proof-of-concept investigation demonstrating that peripherally administered epothilone D can enter the brain and have an effect on neurons. In accordance with established protocols (Carbonell et al., 1998; Spain et al., 2010; Alder et al., 2011) we showed that a single episode of mild TBI generated significant loss of consciousness, as measured by injury-induced apnoea and delayed righting, in the absence of overt macroscopic change. At the cellular level there was no significant loss of YFP+ neurons, axons or dendrites or dendritic spines and no evidence of neuronal atrophy by 7 days post injury. Moreover, the dose of epothilone D was well tolerated and there were no overt indications of ill health or drug-induced cellular degeneration.

Dendritic spine loss is a characteristic feature of a variety of diseases and injuries of the nervous system (Fiala et al., 2002a; Campbell et al., 2012). Moreover, synapse loss is a likely key candidate for the emergence of functional deficits following TBI. Correct functioning of the neuronal cytoskeleton is crucial for the maintenance of neuronal integrity. Actin has a well-established and integral role in the function of dendritic spines (Matus, 2000; Hotulainen and Hoogenraad, 2010; Lei et al., 2016). More recently, microtubules have been implicated in spine formation and maintenance (Gu et al., 2008; Jaworski et al., 2009; Kapitein et al., 2010; Dent, 2016). To determine the effect of microtubule stabilization at the level of dendritic spines, and to avoid any potential confounding effects of the craniotomy, we examined spines on the radially projecting branches, residing in layer 4/5, of layer 5 YFP+ cortical projection neuron apical dendrites. Interestingly, while filopodia remained unaltered in terms of both length and density, epothilone D had dramatic effects on the spine population, causing an overall reduction in spine length, a shift to a greater proportion of short spines, and an increase in the formation of mushroom spines. The increase in spine density, specifically of mushroom spines, was an unanticipated response to epothilone D treatment and was observed both within the brain-injured and sham-operated cortex. This may indicate a more generalized effect of microtubule stabilization at the subtle level of the dendritic spine, rather than a neuroprotective effect of the drug evoked by injury. It is notable that the increase in spine density occurred specifically in the mushroom spines, which have previously been shown to be a relatively stable population likely to host synapses (Hering and Sheng, 2001; Fiala et al., 2002b). Although we were unable to trace axons to determine innervation interactions between degenerating axons and newly formed spines or determine the functionality of the newly formed spines using the current methodology, future studies quantifying colocalization of synaptic markers, ultrastructural and electorphysiological analysis as well as *in vivo* imaging of synaptic turnover could be used to determine the presense/absence and functionality of synapses on newly formed spines and validate the therapeutic potential of epothilone D.

Although the current study did not directly address the mechanism by which epothilone D increased spine density, it is plausibile that this may be due to a direct effect on the spines themselves, the axons innervating the spines or a more indirect effect. Nonetheless, in terms of therapeutic potential, microtubule stabilization may be useful for preserving spine and synapse integrity following injury. In accordance with this, we revealed a distinct increase in spine density following epothilone treatment. Moreover, spine restoration has been demonstrated following epothilone D treatment in an Alzheimer’s disease model (Penazzi et al., 2016). Although further investigations into the mechanisms of spine generation and maintenance by epothilone D are required, we postulate that microtubule stabilization and polymerization with epothilone D may prevent microtubule disassembly and catastrophe within both the dendrite shaft and spines, preserving spine integrity and reducing spine loss. If spine generation continues, this may contribute to an overall increase in spine density. Whether this is a detrimental or beneficial response in the aftermath of mTBI remains to be elucidated. Interestingly, since changes in spine morphology have been linked to alterations in synaptic strength and implicated in learning and memory (Bourne and Harris, 2007; Gu et al., 2008; Jaworski et al., 2009), manipulation of microtubule dynamics could potentially be used to modulate these parameters. As the epothilone D induced increase in spine density was unanticipated, based on previous literature future studies should determine whether these alterations evoke measureable functional responses in terms of electrophysiological and functional output.

Mild TBI has been described as a progressive disorder. Although we did not see a significant loss of axons, dendrites or spines by 7 days post-injury in the current study, there was certainly observable axonal damage throughout the ipsilateral cortex and white matter. Interestingly, the epothilone D treatment regime used in the current study did not have a protective effect on degenerating axons and our previous study using a mouse model of amyotrophic lateral sclerosis showed chronic epothilone D exposure may be detrimental to axons (Clark et al., 2018). However, using an *in vitro* model of structural axonal injury we have previously shown that low dose epothilone D has a protective effect on injured axons by increasing injury-induced axonal sprouting (Brizuela et al., 2015), indicating that microtubule stabilization within damaged axons may be partially responsible for the previously described neuroprotective effect of epothilone D and may have feed forward effects with regard to dendritic spine innervation. If followed to later post-injury time points, it is possible we may have observed evidence of neuronal atrophy, progressive axonal degeneration and spine loss following mild lateral FPI, as has been shown in other studies of brain injury (Fiala et al., 2002a; Chen et al., 2010; Gao and Chen, 2011; Gao et al., 2011; Greer et al., 2011; Campbell et al., 2012; Johnson et al., 2012b; Winston et al., 2013) and the previously described therapeutic effects of epothilone D may have been revealed. In our hands, and in accordance with previous studies, mild lateral FPI generated widespread traumatic axonal injury, which was particularly evident in the external and internal capsule white matter tracts (Smith and Meaney, 2000; Spain et al., 2010; Wang and Ma, 2010; Ekmark-Lewen et al., 2013; Smith et al., 2013; Hill et al., 2016). This included both beaded, degenerating axons and degenerated, dystrophic axonal fragments/bulbs. Both phenotypes have been reported in the literature and may represent stages of degeneration, differences in vulnerability between different axonal sub-classes or brain regions, or be specific to the mechanical forces sustained during injury (Reeves et al., 2005; Browne et al., 2011; Hånell et al., 2014).

Interestingly, axonal sprouting has been observed in certain classes of damaged axons in experimental models of brain injury (Salin et al., 1995; Batchelor et al., 2002; Deller et al., 2006; Dickson et al., 2007; Blizzard et al., 2011; Greer et al., 2011). Although not observed in the current study, it is possible that epothilone D could be used to modulate this response. Indeed, using an *in vitro* model, we have shown that epothilone D has dose-dependent effects on regenerating cortical neurons (Brizuela et al., 2015). This concept has important implications for dose-dependent modulation of axonal sprouting *in vivo*, for example enhancing adaptive regenerative attempts or dampening maladaptive sprouting, which may underlie the development of epileptic activity in the aftermath of injury (Santhakumar et al., 2001; Kharatishvili et al., 2006; Bolkvadze and Pitkänen, 2012).

In response to FPI we observed mild ipsilateral astroglial activation in the absence of glial scar formation. Contrary to previous studies, astroglial activation was not influenced by microtubule stabilization in the current study (Hellal et al., 2011; Popovich et al., 2014; Ruschel et al., 2015). This may have been due to a range of factors including the type of agent used (a taxane versus an epothilone), the concentration or timing of the epothilone D dose, the mild nature of the glial activation, or the context of the injury (for example, brain versus spinal cord). The absence of an observable injury-induced alteration in dendritic spine density in the current study may have been due to the spine population investigated, the proximity of the spines to the site of impact, the brain region assessed and its proximity to the injury, as well as the time point at which analysis was performed. Therefore, to elucidate the efficacy of microtubule stabilization as a therapeutic intervention for mTBI further investigations will be required to reveal the full repertoire of effects of microtubule stabilization in both the acute and chronic phases of the injury response. Future studies will use a range of dose regimes, including various concentrations and times of administration throughout the post-injury sequalae, to capture the dynamic aspects of injury-induced degeneration and remodeling.

In summary, our findings indicate that peripherally administered microtubule-stabilizing drugs alter synaptic plasticity at the level of the dendritic spine. This has important implications for controlling neuroplasticity in the aftermath of brain injury. More generally, microtubule-stabilizing agents may be useful for manipulating various aspects of the neuro-glial response to injury and disease, as well as providing a modulatory tool for investigating microtubule dynamics *per se*. Due to the ubiquity of microtubules it will be imperative to consider the full repertoire of roles in which they are involved, and take into consideration the interplay between intrinsic factors such as neuronal class and age and extrinsic factors such as the type and severity of injury.

## Author Contributions

All authors have made a substantial contribution to the work and approved it for publication. Specifically JC, TD, CB conceived and designed the experiments, wrote the manuscript and analyzed and interpreted the data. JC, ZZ, MB, and KL performed the experiments and acquired the data.

## Conflict of Interest Statement

The authors declare that the research was conducted in the absence of any commercial or financial relationships that could be construed as a potential conflict of interest.
